# Emergency Excision of Cardiac Myxoma and Endovascular Coiling of Intracranial Aneurysm after Cerebral Infarction

**DOI:** 10.1155/2013/839270

**Published:** 2013-09-16

**Authors:** Youssef Al-Said, Heyam Al-Rached, Saleh Baeesa, Khalil Kurdi, Ibrahim Zabani, Ahmed Hassan

**Affiliations:** ^1^Neurosciences Department, King Faisal Specialist Hospital and Research Center, Jeddah 21499, Saudi Arabia; ^2^Radiology Department, King Faisal Specialist Hospital and Research Center, Jeddah 21499, Saudi Arabia; ^3^Anesthesia Department, King Faisal Specialist Hospital and Research Center, Jeddah 21499, Saudi Arabia

## Abstract

Cardiac myxoma is the most common primary tumor of the heart, located mainly in the left atrium. Cerebral embolization or intracranial aneurysm formation as a consequence of left atrial myxomas has been well documented, whereas myxoma embolization causing the combination of cerebral infarction and intracranial myxomatous aneurysm is rare. We report herein, a 67-year-old female with a cardiac myxoma who experienced a left hemispheric embolic ischemic stroke and in addition was found to have right internal carotid artery aneurysm. The patient underwent emergency surgical excision of left atrial myxoma 2 hours after the stroke onset and endovascular coiling of the aneurysm a week later. Although the timing of cardiac surgery is controversial in patients who have had recent ischemic stroke, we recommend immediate resection of cardiac myxoma, if feasible, and early endovascular treatment of associated intracranial myxomatous aneurysms.

## 1. Introduction

Cardiac myxoma, occurring at an incidence of 0.002% among general population, is the most common heart tumor, representing 50% of all cardiac tumors, and about 75% of them are located in the left atrium [1]. Neurologic complications resulting from cardiac myxoma are seen in 20 to 35% of patients because of their predilection to embolize [2, 3]. In at least half of the cases, cerebral arteries are affected, leading to an embolic ischemic stroke [3]. In contrast, the formation of intracranial aneurysms associated with left atrial myxoma is a less common phenomenon [4, 5].

The natural history and the pathogenesis of myxomatous aneurysms are not well studied. There is no gold standard for the therapy of myxoma-related cerebral aneurysms. Surgical resection of myxoma usually relieves any recurrence of the neurological symptoms. However, open-heart surgery requires anticoagulation for cardiopulmonary bypass, and patients who have suffered a recent preoperative stroke are at increased risk for hemorrhagic conversion [6]. There are no clear guidelines recommending the optimal interval from the onset of stroke to the time of the surgery [2, 4].

Herein, we present an unusual case of a 67-year-old female with left atrial myxoma who developed both a right intracranial carotid artery myxomatous aneurysm and an acute left middle cerebral arterial infarction. She underwent immediate excision after stroke of the myxoma with no major enlargement of the infarct or hemorrhagic transformation. Endovascular treatment of the aneurysm was done a week later.

## 2. Case Report

**Figure 1:**
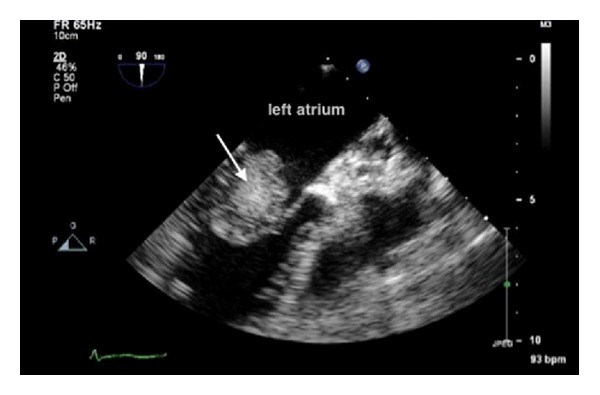
Echocardiogram showing left atrial myxoma (arrow).

A 67-year-old female was referred to our institution after investigating her 6-month history of dyspnea and dizziness, which was attributed to a cardiac tumor. Her physical examination, on admission for cardiac surgery, revealed normal vital signs; cardiac auscultation revealed a 3/6 systolic murmur. Her neurological examination was normal. Routine laboratory investigations and chest radiograph were within the normal. Electrocardiogram (ECG) revealed sinus rhythm. Transthoracic 2-D echocardiography revealed a left atrial tumor attached to the atrial septum causing mitral and tricuspid valves prolapse; there was no associated thrombus (Figure 1).

**Figure 2:**
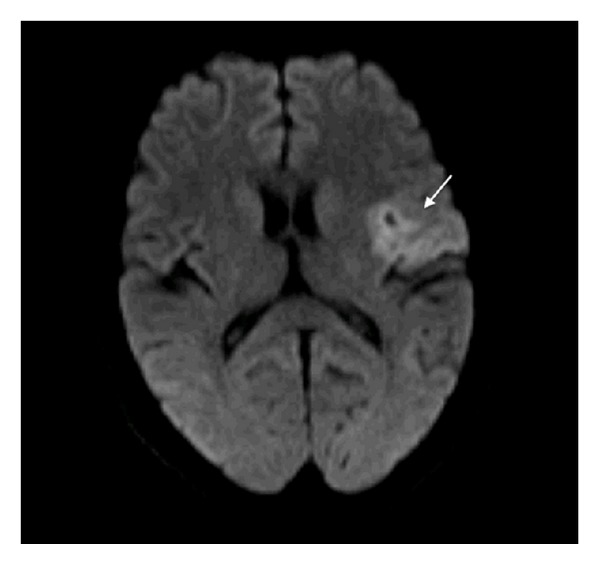
Axial diffusion MRI scan demonstrating high intensity lesion at the left frontal region consistent with acute infarction.

**Figure 3:**
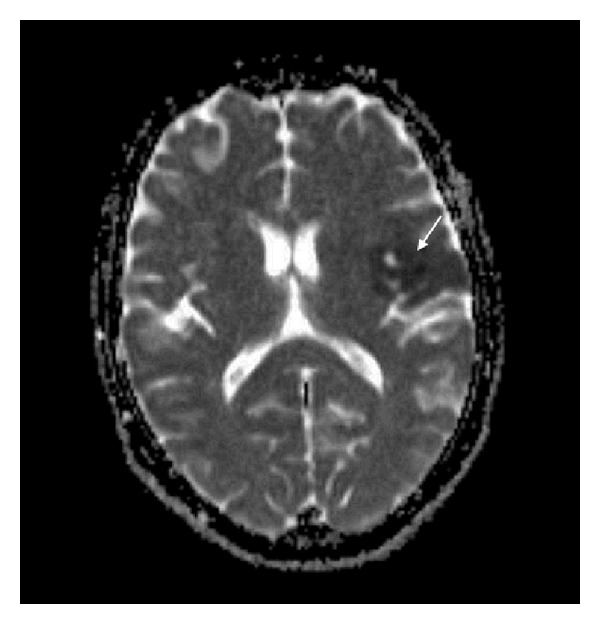
Axial ADC-MRI scan demonstrating low intensity lesion at the left frontal region consistent with acute infarction.

**Figure 4:**
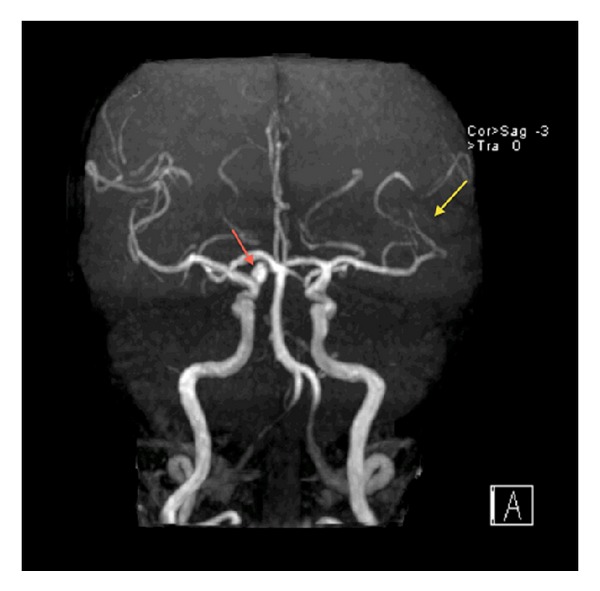
TOF-MRA scan in coronal view demonstrating cut-off at left upper division of MCA (yellow arrow). There is right ICA aneurysm (red arrow).

The decision was made for operative intervention and informed consent was obtained, but unfortunately early morning of the day of the surgery, she suddenly developed aphasia and right-sided hemiplegia. At the time of the event, her vital signs were blood pressure 130/70 mmHg, pulse rate 81 beats/minute, respiratory rate 18 breaths/minute, and body temperature 36.6°C orally. Neurological examination showed that the patient was alert and oriented. She had mild expressive aphasia, right hemianopia, right central facial palsy, right hemiplegia, and extensor plantar reflex on the right. NIH Stroke Scale (NIHSS) score was 12 and the Glasgow coma score 14. The ECG showed normal sinus rhythm with no ST-T abnormalities. Blood laboratory data revealed unremarkable abnormalities particularly for coagulopathy screening. Immediate computed tomography (CT) scan of the brain revealed no hemorrhage or a left hemispheric infarct. An urgent magnetic resonance imaging (MRI) of the brain utilizing a stroke protocol demonstrated an acute infarction in the distribution of the left middle cerebral artery (Figures 2 and 3). In addition a right paraophthalmic internal carotid artery (ICA) aneurysm measuring 8 mm in maximal diameter was demonstrated on magnetic resonance angiography (MRA). Cut-off at the frontal branches of the left middle cerebral artery from cardiac embolization was also seen (Figure 4). The carotid and vertebrobasilar vessels were normal.

The first day of infarction poses the least risk of hemorrhagic transformation therefore emergent cardiac surgery was recommended. Intravenous thrombolysis with rTPA was not recommended because the etiology of the infarction was most probably embolization of the myxoma. The findings, recommendations, and options were discussed with the patient and her family, the decision was made for emergency excision of cardiac tumor followed by endovascular treatment of ICA aneurysm, and informed consent was obtained.

**Figure 5:**
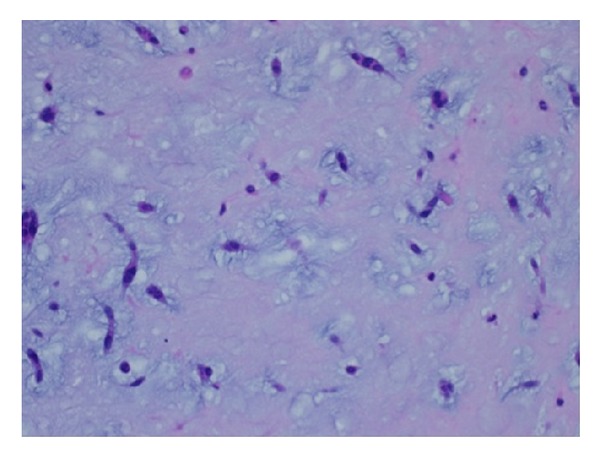
Microphotograph of the surgical specimen showing spindle and stellate myxoma cells seen within myxoid stroma (hematoxylin and eosin, original magnification ×400).

The patient underwent resection of the left atrial mass under cardiopulmonary bypass and reconstruction of the atrial septum using a bovine pericardial patch. The diagnosis of benign atrial myxoma was confirmed by histopathological examination without overlying thrombus (Figure 5).

**Figure 6:**
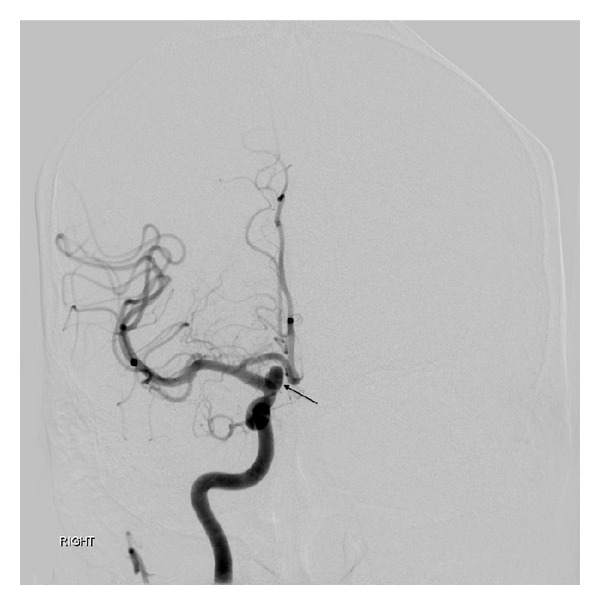
Anterior-posterior view of right carotid angiogram demonstrating paraophthalmic ICA aneurysm (arrow).

**Figure 7:**
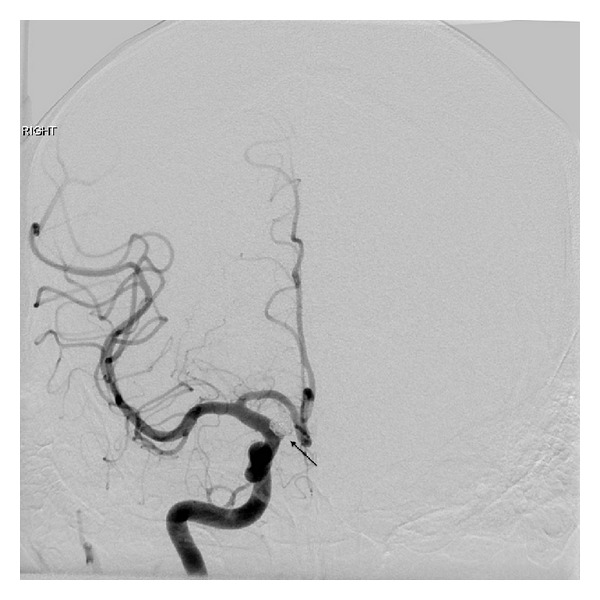
Postendovascular coiling of ICA aneurysm angiogram (arrow) demonstrating complete obliteration.

The patient had uneventfully recovered from cardiac surgery, and her postoperative general and neurological course was stable. A 24 h followup CT of the brain and echocardiography showed no significant enlargement of the infarct or hemorrhagic transformation and any residual myxoma and mitral/tricuspid regurgitation, respectively. After one week, she underwent cerebral angiogram that visualized the aneurysm (Figure 6), and endovascular coiling of ICA aneurysm was performed with complete obliteration (Figure 7).

**Figure 8:**
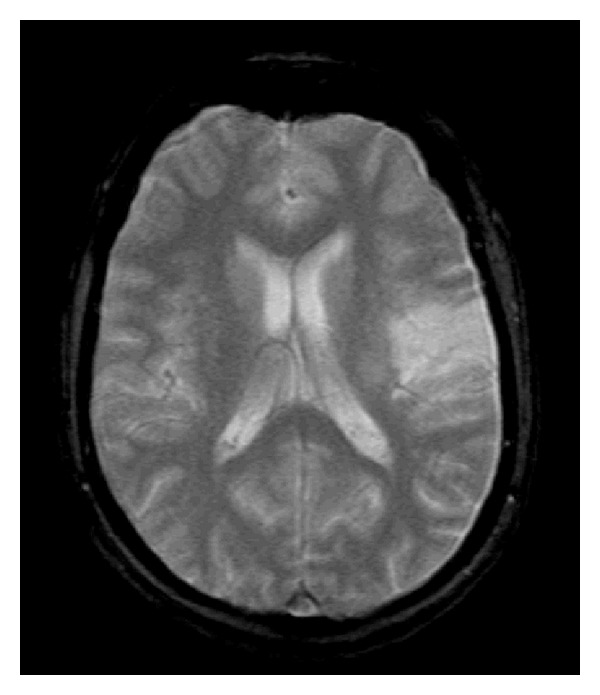
T2WI-MRI scan after one year demonstrating regression of the previous infarction.

**Figure 9:**
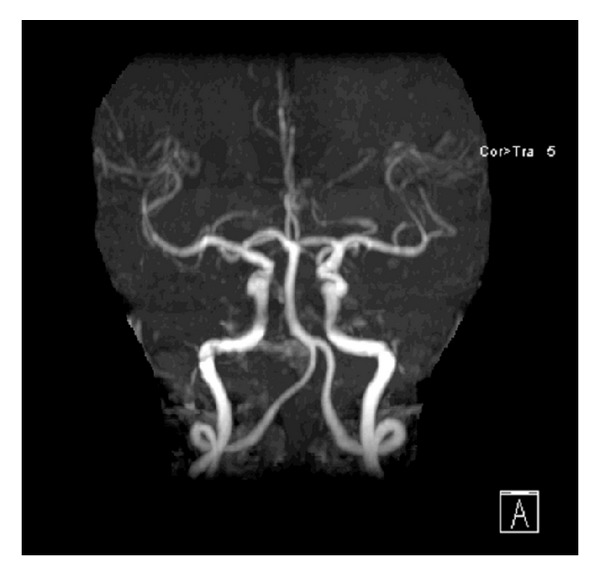
TOF-MRA scan after one year demonstrating persistent obliteration of coiled ICA aneurysm.

The patient was discharged on 2 antiplatelet therapies and received outpatient speech and physical therapy. At one-year followup, she had marked recovery of her weakness and aphasia: MRI and MRA (Figures 8 and 9) revealed regression of stroke size and a completely obliterated ICA aneurysm.

## 3. Discussion

Neurological manifestations are one of the most common serious presentations of cardiac myxoma that occur up to 30% of patient [1, 3]. Ischemic cerebral infarction is the most common neurologic complication, occurring mainly in the left middle cerebral artery territory [2]. A recent study reported that embolic stroke was observed in 9–22% of atrial myxomas [3, 7]. The incidence of embolization is not related to tumor size [3, 7] but instead is related to the mobility and friability of the tumor [3, 4, 7–9].

There are no clear guidelines for the immediate medical management following stroke from atrial myxoma. Anticoagulants and antiplatelet agents are used with the presumption that some of the embolic component is a thrombus but may not be protective [10]. There is a significant body of literature that recommends treating any cardiac myxoma soon after diagnosis to improve cardiac function and prevent systemic embolization. Resection of an atrial myxoma without a recent stroke is relatively simple operation. However, the removal of the myxoma in the setting of a recent stroke poses a difficult management problem. Systemic anticoagulation required for cardiopulmonary bypass may become an issue in the patient who has suffered a recent stroke. A large embolic stroke may become hemorrhagic and may extend brain damage, especially during the first week after stroke [11]. There are no clear guidelines in the literature indicating the safe interval from the onset of stroke to the time of the surgery [2, 4], so timing of surgery is still controversial and needs to be clarified [2]. In a previous report of left atrial myxoma presented as stroke, the surgery was done after a waiting time of 4 weeks [6]. Due to the immediate diagnosis of stroke, the presence of myxomatous intracranial aneurysm, and because the risk of hemorrhagic transformation is the least in the first day of infarction and the increased risk of recurrent ischemic stroke with delayed surgery, the patient was taken for emergency cardiac surgery within 2 hours of the stroke.

Another neurological complications of myxoma are parenchymal brain metastases and the formation of intracranial aneurysms, which may rupture causing intracerebral or subarachnoidal hemorrhage [3, 12].

To our knowledge, about forty cases of intracranial aneurysms associated with myxoma have been reported in the literature [13]. Intracranial aneurysms are rare complication of myxomatous emboli [3]. Aneurysm formation associated with embolized atrial myxoma is not caused by blood-flow dynamics but rather by myxomatous tumor invasion into the vessel wall [13–16]. Our patient has an isolated intracranial aneurysm, located in the right paraophthalmic internal carotid artery.

The natural history of myxomatous aneurysms is not well characterized, which further complicates treatment. Resolution after surgical removal of myxoma, potential progressive enlargement with possible hemorrhage, and spontaneous resolution or stabilization has been reported [17, 18].

The limited experience in treating small numbers of patients with this complication is the reason why treatment of intracranial aneurysms associated with cardiac myxoma is not precisely defined. There are no evidence-based guidelines that defined when cerebral angiography or aneurysm treatment should be conducted in a patient with cerebrovascular manifestation of atrial myxoma. Our patient underwent cerebral angiography and successful coiling of the aneurysm one week after surgery.

 Appropriate and complete resection of myxoma is the method of choice because it minimizes the risk of tumor embolization, but it does not eliminate the risk of delayed aneurysm formation regardless the mechanism of development either by embolization or metastasis [13, 19]. For this reason, follow-up monitoring for the development of aneurysms, using noninvasive imaging (MRI/MRA) is recommended after intervention for myxoma resection [12].

For our patient, we repeated the cerebral angiography one year after aneurysm coiling. It did not show aneurysm enlargement, and no other aneurysm was detected.

## 4. Conclusion

Cardiac myxoma has high tendency to produce disabling neurological complications due to the risk of embolic episodes, which emphasizes the need for its prompt surgical excision as soon as the diagnosis is confirmed. Timing of surgery is still controversial in patients who have had recent neurological insults. We recommend urgent surgical resection of the tumor, if feasible, and early screening and management of associated cerebral aneurysms.
